# Fabrication, Structure, and Properties of Nonwoven Silk Fabrics Prepared with Different Cocoon Layers

**DOI:** 10.3390/ijms241411485

**Published:** 2023-07-14

**Authors:** Yun Yeong Choi, Mi Jin Jang, Byung-Dae Park, In Chul Um

**Affiliations:** 1 Department of Biofibers and Biomaterials Science, Kyungpook National University, Daegu 41566, Republic of Korea; 2 Preclinical Research Center, Daegu-Gyeongbuk Medical Innovation Foundation, Daegu 41061, Republic of Korea; 3 Department of Wood and Paper Science, Kyungpook National University, Daegu 41566, Republic of Korea

**Keywords:** silk web, nonwoven silk fabric, silkworm cocoon layer, structural characteristics, mechanical properties, cell viability

## Abstract

In this study, five different nonwoven silk fabrics were fabricated with silk fibers from different cocoon layers, and the effect of the cocoon layer on the structural characteristics and properties of the nonwoven silk fabric was examined. The diameter of the silk fiber and thickness of the nonwoven silk fabric decreased from the outer to the inner cocoon layer. More amino acids with higher hydrophilicity (serine, aspartic acid, and glutamic acid) and lower hydrophilicity (glycine and alanine) were observed in the outer layers. From the outer to the inner layer, the overall crystallinity and contact angle of the nonwoven silk fabric increased, whereas its yellowness index, moisture retention, and mechanical properties decreased. Regardless of the cocoon layer at which the fiber was sourced, the thermal stability of fibroin and sericin and good cell viability remained unchanged. The results of this study indicate that the properties of nonwoven silk fabric can be controlled by choosing silk fibers from the appropriate cocoon layers. Moreover, the findings in this study will increase the applicability of nonwoven silk fabric in the biomedical and cosmetic fields, which require specific properties for industrialization.

## 1. Introduction

Silk is a naturally occurring biomaterial consisting of fibroin and sericin and has good biocompatibility [1,2,3], biodegradability [4,5,6], blood compatibility [7,8], good mechanical properties [9], cytocompatibility [10,11,12], and minimal inflammatory reaction to the body [13]. In recent decades, owing to these unique properties, studies on silk have been intensively conducted for various biomedical and cosmetic applications, including wound dressing [14,15,16], membrane for guided bone regeneration [17,18,19], drug delivery [20,21], nerve conduits [22], and facial mask packs [23]. Although various forms of regenerated silk, including electrospun web, film, and gel, have been used for these applications, natural nonwoven silk fabrics have recently attracted researchers’ attention because of their superior mechanical properties, simple fabrication with diverse sizes, and easy mass production.

Natural nonwoven silk fabric is fabricated by reeling silk fibers in a silkworm cocoon, followed by wet and hot press treatments. A silkworm cocoon is composed of different layers [24,25,26,27,28] with different structures and properties. The silk fiber in the outer layer of the cocoon has a larger diameter than that of the inner layer [12]. From the inner layer to the outer layer, the content of sericin increases, and the mechanical properties of the resulting material are also dependent on the layer of the silkworm cocoon [25]. Due to the different structural characteristics and composition of the silkworm cocoon layer, each silkworm cocoon layer has different biomedical performances. Among these, the middle layer has the best performance for guided bone regeneration [27,28].

Various biomedical and cosmetic applications require silk materials with diverse and complicated performances. Therefore, the effect of various preparation conditions, including the press temperature [29], press time [30], reeling bath temperature [31], air-laid method [32], silkworm variety [33], rayon addition [34], and sericin content [35], on the structure and properties of nonwoven silk fabrics has been examined to obtain their properties. However, the effect of the cocoon layer on nonwoven silk fabrics is yet to be examined.

In this study, we fabricated nonwoven silk fabrics using silk fibers from five different cocoon layers. The effect of different cocoon layers on the structural characteristics and properties of nonwoven silk fabric was investigated.

## 2. Results and Discussion

### 2.1. Morphology of Silk Web and Nonwoven Fabric

Table 1 shows images of the silk web and nonwoven fabric prepared with silk fibers from different cocoon layers. Silk web exhibits regularly arranged silk fibers because it was prepared by reeling the silk fibers from the silkworm cocoon using an electric winder. In contrast, the silk fibers in the nonwoven fabric became rugged after wet and hot press treatments. The generation of bent and rugged silk fibers in nonwoven fabric is attributed to the fast removal of water during the wet and hot press treatments [31,35]. From the 1st layer (outermost layer) to the 5th layer (innermost layer), the silk fiber in the silk web becomes thinner; that is, the diameter of 33 ± 4.7 μm for the silk fiber in the 1st layer decreased to 25 ± 2.9 μm in the 5th layer. This is ascribed to the decrease in the diameter of the silk fiber of the silkworm cocoon from the outer to the inner layer of the cocoon [12].

**Figure 1:**
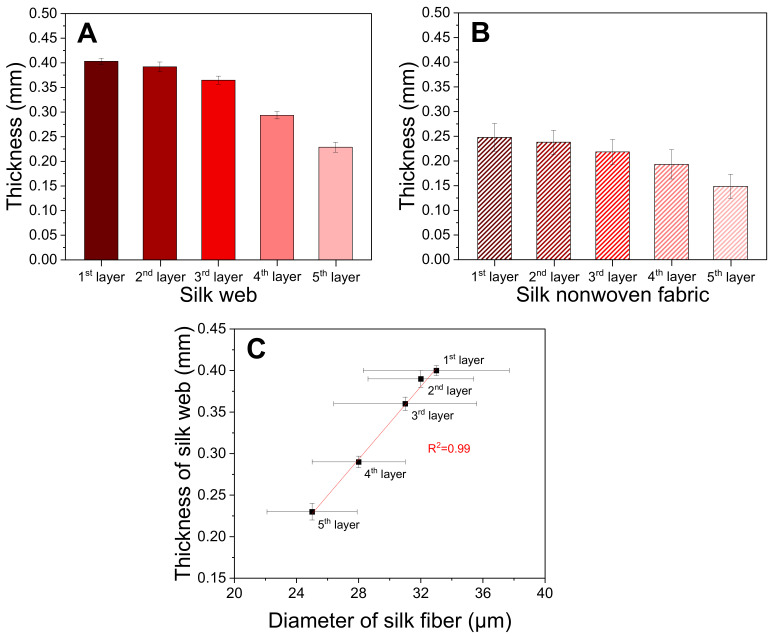
Thickness of the (**A**) silk web and (**B**) nonwoven silk fabric prepared using fibers from different silk cocoon layers (*n* = 21). (**C**) Relationship between the diameter of the silk filament (*n* = 50) and thickness of the silk web (*n* = 21).

Figure 1A,B show the thicknesses of the silk web and nonwoven fabric prepared using fibers from different cocoon layers, respectively. The thickness of the silk web from the 1st layer is 0.40 mm, which decreases to 0.23 mm in the 5th layer. The thickness of the nonwoven silk fabric using fibers from the 1st layer is 0.25 mm, which decreases to 0.15 mm in the 5th layer. Considering a constant reeling time (i.e., length of the silk fiber) for all silk webs and nonwoven fabrics, the decrease in the thickness of the silk web and nonwoven fabric from the 1st layer to the 5th layer is attributed to the decrease in the fiber diameter. Figure 1C confirms the linear correlation between the diameter of the fiber and thickness of the silk web (*R*^2^ = 0.99). The reduced thickness of the nonwoven silk fabric compared to that of the silk web is attributed to the effect of the compression of the silk web due to the hot press treatment [30,31].

### 2.2. Structural Characteristics of the Silk Web and Nonwoven Fabric

Table 2 shows the amino acid composition of the nonwoven silk fabric prepared using fibers from different cocoon layers. From the outer layer to the inner layer, the glycine and alanine contents increased from 35.92% to 40.30% and from 23.78% to 27.83%, respectively, whereas the serine and aspartic acid contents decreased from 14.19% to 11.36% and from 5.55% to 3.55%, respectively. This is ascribed to the increase in the sericin content from the outer to the inner layer. This result reaffirms previous studies, whereby the sericin content in a cocoon is higher in the outer layers than the inner layers [12,25,36].

**Figure 2:**
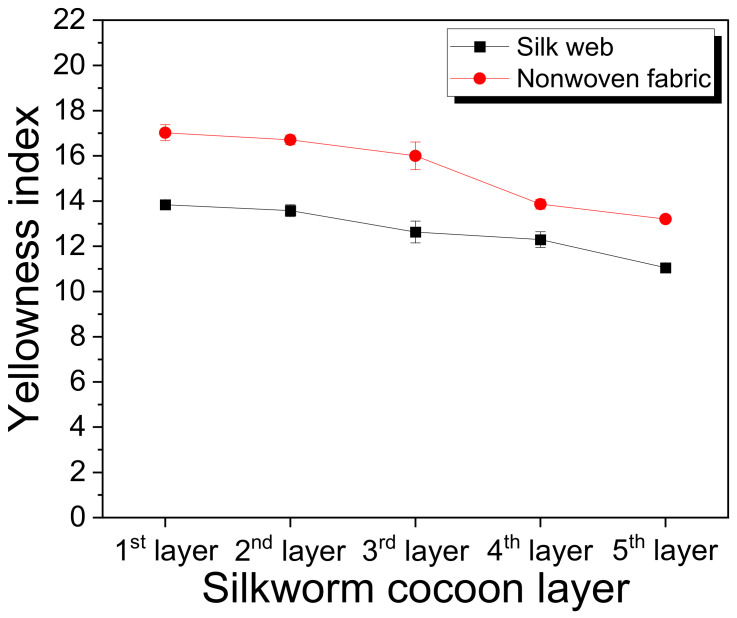
Yellowness index of the silk web and nonwoven silk fabric prepared with fibers from different silkworm cocoon layers (*n* = 3).

To quantitatively examine the color change of the silk web and nonwoven fabric, their yellowness index was measured; the results are shown in Figure 2. The yellowness indexes of the nonwoven silk fabric are higher than those of the silk web. This result is consistent with previous reports [30,32,34,35] and is attributed to the yellowing of silk due to the heat treatment during the preparation of the nonwoven fabric by hot pressing at 200 °C. Setoyama [37] reported that the yellowing of silk is ascribed to the loss of amino acids with the hydroxyl group in silk. For both silk web and nonwoven silk fabric, the yellowness indexes decreased from the outer to the inner layer. Considering the increase in the sericin content in silk from the inner to the outer layer [12,25,36], sericin has a larger contribution to the yellowness of silk than fibroin. Interestingly, the degree of increase of the yellowness index by the hot press treatment (i.e., difference of the yellowness index between the nonwoven silk fabric and silk web) in the silk samples using silk fibers from the 1st, 2nd, and 3rd layers is higher than that from the 4th and 5th layers. This is attributed to the decrease in the sericin content of from the outer to the inner layer (Table 2). In particular, sericin is thermally weaker and has a lower decomposition temperature than fibroin [35,38,39] and higher amino acid content with the hydroxyl group (especially serine), which is responsible for its higher influence in the yellowing of the nonwoven silk fabric than fibroin [40].

**Figure 3:**
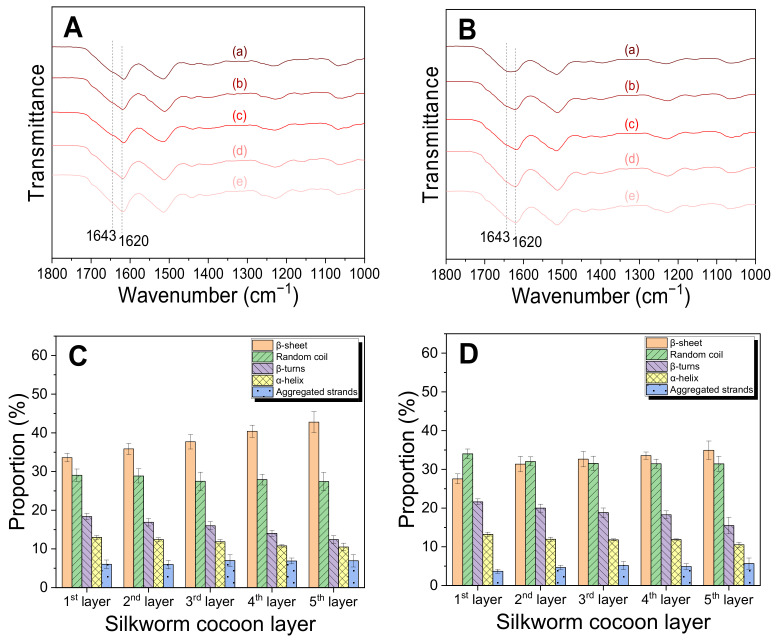
ATR–FTIR spectra and proportion of molecular conformation of the (**A**,**C**) silk web and (**B**,**D**) nonwoven silk fabric prepared using fibers from different silkworm cocoon layers (*n* = 7); (a) 1st layer, (b) 2nd layer, (c) 3rd layer, (d) 4th layer, and (e) 5th layer.

The crystallinity of silk determines its mechanical properties [41,42,43,44], post-drawing performance of its wet spun fiber [41,45], and its moisture retention [42]. Therefore, the molecular conformation and crystallinity of silk have been investigated using Fourier transform infrared (FTIR) spectroscopy [46,47]. In this study, FTIR was conducted on the silk web and nonwoven fabric prepared with fibers from different cocoon layers; the results are shown in Figure 3.

All silk webs (Figure 3A) exhibit an IR absorption peak at 1620 cm^−1^, which is attributed to the β-sheet crystallite [30,48,49,50,51]. For the nonwoven silk fabric (Figure 3B), the IR peak at 1620 cm^−1^ and shoulder at 1643 cm^−1^ are attributed to the β-sheet crystallite and random coil conformation, respectively. This implies the higher random coil conformation in the nonwoven silk fabrics due to the disruption of the β-sheet crystallite by the hot press treatment at 200 °C [30,31,33,35,52].

The proportion of molecular conformation of silk was calculated using the deconvolution of the FTIR spectra to quantitatively examine the conformational change of the silk web and nonwoven silk fabric prepared using fibers from different cocoon layers. For both the silk web and nonwoven silk fabric (Figure 3C,D), the proportion of β-sheet conformation increased and that of the random coil decreased from the outer layer to the inner layer. This indicates that the inner layer contains more β-sheet crystallite in both the silk web and nonwoven silk fabric because of the (1) larger amount of silk fibroin in the inner layer than that of the outer layer [36], as shown in Table 2, and (2) larger amount of β-sheet crystallite in the silk fibroin than that in silk sericin (i.e., silk fibroin is more crystallized than sericin) [12,35,53]. The silk webs (33.6–42.8%) have higher β-sheet crystallite content than that of the nonwoven silk fabrics (27.5–34.9%), which is consistent with the result of previous reports [35,52]. Lee et al. reported that attenuated total reflectance (ATR)–FTIR reflects that the sample surface and β-sheet crystallite of sericin in silk are disrupted by the hot press treatment at 200 °C [35].

**Figure 4:**
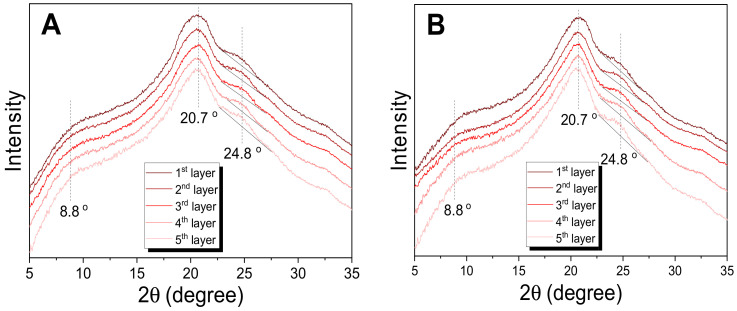
XRD results of the (**A**) silk web and (**B**) nonwoven silk fabric prepared using fibers from different silkworm cocoon layers.

Unlike the ATR–FTIR technique that reflects the silk surface (i.e., sericin), X-ray diffraction (XRD) detects the entire sample by X-ray penetration of the entire sample. Therefore, XRD measurement can be used as a complementary tool to examine the microstructure of silk. In this study, XRD measurement was conducted on the silk web and nonwoven silk fabric prepared with fibers from different cocoon layers; the results are shown in Figure 4.

Regardless of the cocoon layer, all silk webs and nonwoven silk fabrics have three XRD peaks at 2θ = 8.8°, 20.7°, and 24.8° attributed to the β-sheet crystallite [12,31,43,45,53,54]. The XRD peak of the nonwoven silk fabrics (Figure 4B) at 20.7° has higher intensity than that of the silk webs (Figure 4A), implying the higher crystallinity of the nonwoven silk fabrics than that of the silk webs. The higher crystallinity of the nonwoven silk fabric from the XRD measurement is contrary to the results of the FTIR measurement, which is ascribed to their different detection methods [31]. As mentioned above, ATR–FTIR is focused on the surface of the silk sample (sericin), whereas XRD detects the entire sample (fibroin and sericin). Therefore, the ATR–FTIR results achieved a lower proportion of β-sheet crystallite in the nonwoven silk fabric than that of the silk web because of the disruption of the sericin crystallite during the hot press method. As the thermally induced crystallization of silk fibroin by the hot press process at 200 °C [55,56] overcomes the disruption of the sericin crystallite [30,31,33,35,35,52], the overall crystallinity of the nonwoven silk fabric is higher than that of the silk web.

The results of previous studies [31,53] revealed the increased intensity of the XRD peak at 24.5° when the crystallinity of the silk samples increases, indicating that this diffraction peak can be utilized as a barometer to detect the changes in the crystallinity of the silk samples. The XRD peak at 24.8° of the silk web and nonwoven silk fabric prepared with fibers from the 5th cocoon layer was more evident than that prepared with the fibers from the 1st cocoon layer, indicating the higher crystallinity of the silk web and nonwoven silk fabric obtained using the 5th layer. As mentioned above, this is attributed to (1) the higher silk fibroin content in the 5th layer than that in the 1st layer and (2) higher crystallinity of the silk fibroin than that of sericin.

Table 3 shows the XRD patterns of the silk web and nonwoven silk fabric prepared with fibers from different cocoon layers. The silk webs have four XRD spots at 2θ of 20.7° and 24.8°, which are ascribed to the straight arrangement of the silk fibers with a cross angle of 30°. However, for the nonwoven silk fabrics, the spots are blunt and arc-shaped because the straight arrangement of the web is bent during the wet and hot press treatments for the preparation of nonwoven silk fabric [31,35], as shown in Table 1.

**Figure 5:**
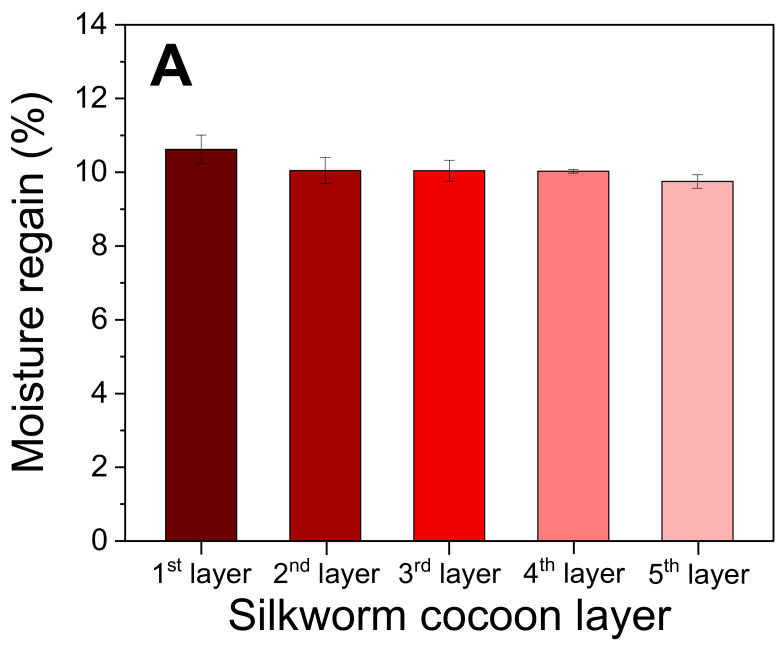

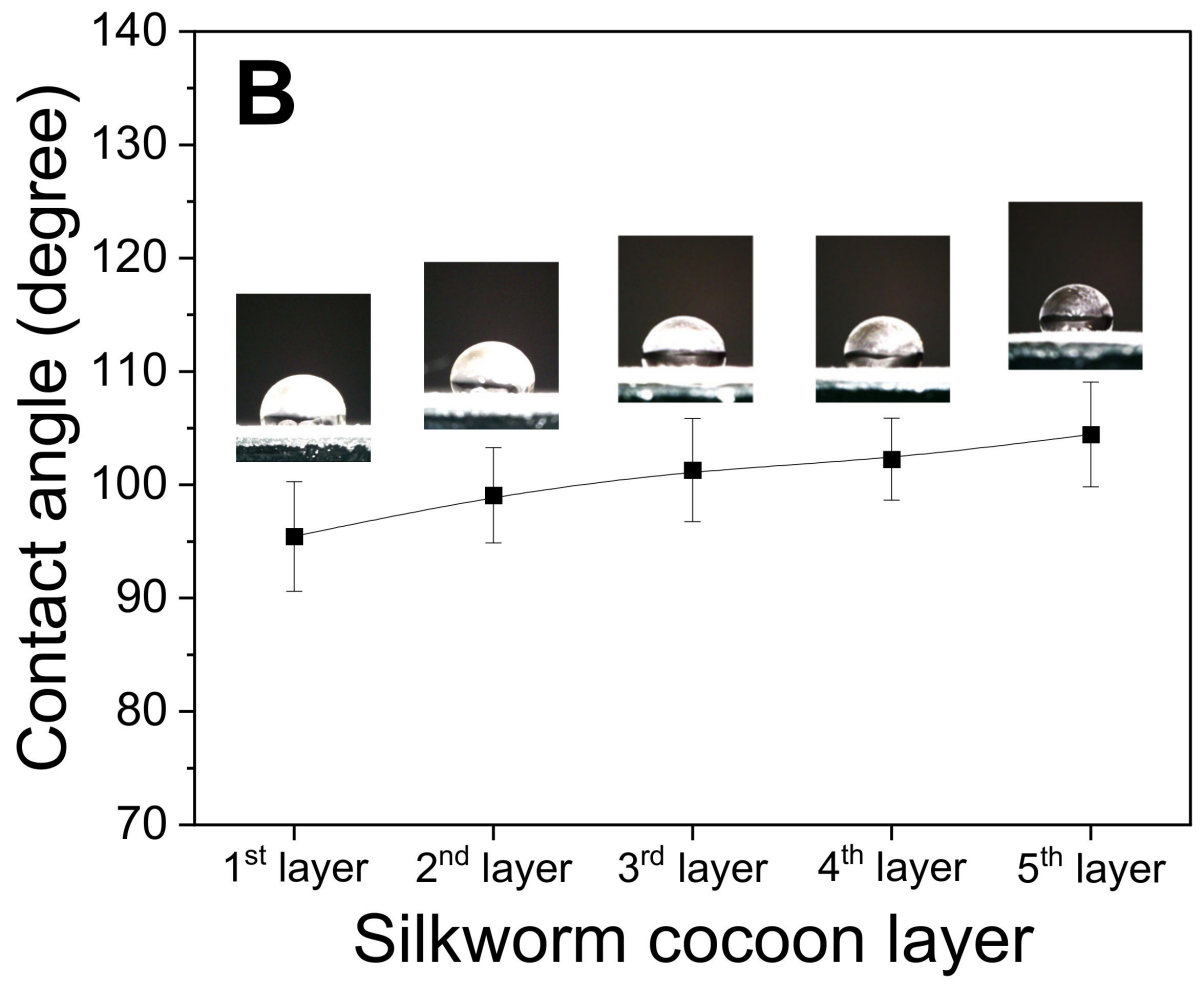
(**A**) Moisture retention of the silk web (*n* = 3) and (**B**) contact angle of the nonwoven silk fabric (*n* = 7) prepared using fibers from different silk cocoon layers.

The water absorption ability and hydrophilicity of a material indicate its performance for biomedical and cosmetic applications [14,15,16,23,33,34,50]. Therefore, the water retention and contact angle of the silk samples prepared with fibers from different cocoon layers were measured; the results are shown in Figure 5. The silk web prepared with the fibers from the 1st layer has a moisture retention of 10.6%, which decreased as the layer number is increased (Figure 5A). The contact angle of the nonwoven silk fabric increases from the outermost to the innermost layer (Figure 5B). These results indicate that the hydrophilicity of the silk web and nonwoven silk fabric decreases from the outer layer (1st layer) to the inner layer (5th layer), which is related to the compositional change of the cocoon layer. In particular, from the outer layer to the inner layer, the silk fibroin content increases, and that of sericin decreases. As sericin is more hydrophilic [57] and less crystalline than silk fibroin [12,35,53], it allows easier access to water, resulting in its higher moisture retention and lower contact angle. As a result, the silk web prepared with the fibers from the 1st layer (higher sericin content) has higher moisture retention and lower contact angle than that prepared with the fibers from the 5th layer (lower sericin content).

### 2.3. Thermal and Mechanical Properties of the Silk Web and Nonwoven Silk Fabric

**Figure 6:**
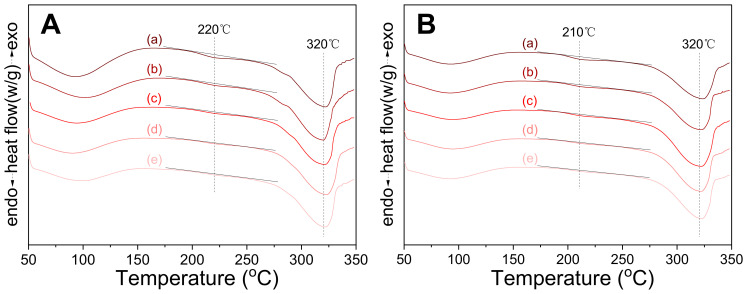
DSC thermograms of the (**A**) silk web and (**B**) nonwoven silk fabric prepared using fibers from different cocoon layers; (a) 1st layer, (b) 2nd layer, (c) 3rd layer, (d) 4th layer, and (e) 5th layer.

To examine the thermal behavior of the silk web and nonwoven silk fabric, differential scanning calorimetry (DSC) was conducted; the result is shown in Figure 6. The silk web exhibits a small and broad endothermic peak at approximately 220 °C and another peak at approximately 320 °C, which are attributed to the thermal decomposition of sericin [58] and silk fibroin [56], respectively. For the nonwoven silk fabric, the endothermic peak corresponding to sericin shifted to 210 °C. This can be ascribed to the disruption of the crystalline of sericin by the hot press treatment (Figure 3) [30,31,33,35,52] owing to the considerable effects of crystallinity on the thermal decomposition temperature of silk [59].

The cocoon layer has no considerable influence on the thermal decomposition temperatures of sericin and silk fibroin for both the silk web and nonwoven silk fabric. Interestingly, the endothermic peaks at 210 °C (nonwoven silk fabric) and 220 °C (silk web) decreases using fibers from the outermost to the innermost layer. Considering the decrease in the sericin content of sericin from the outermost to the innermost layer, the decrease in the peak size can be easily rationalized.

**Figure 7:**
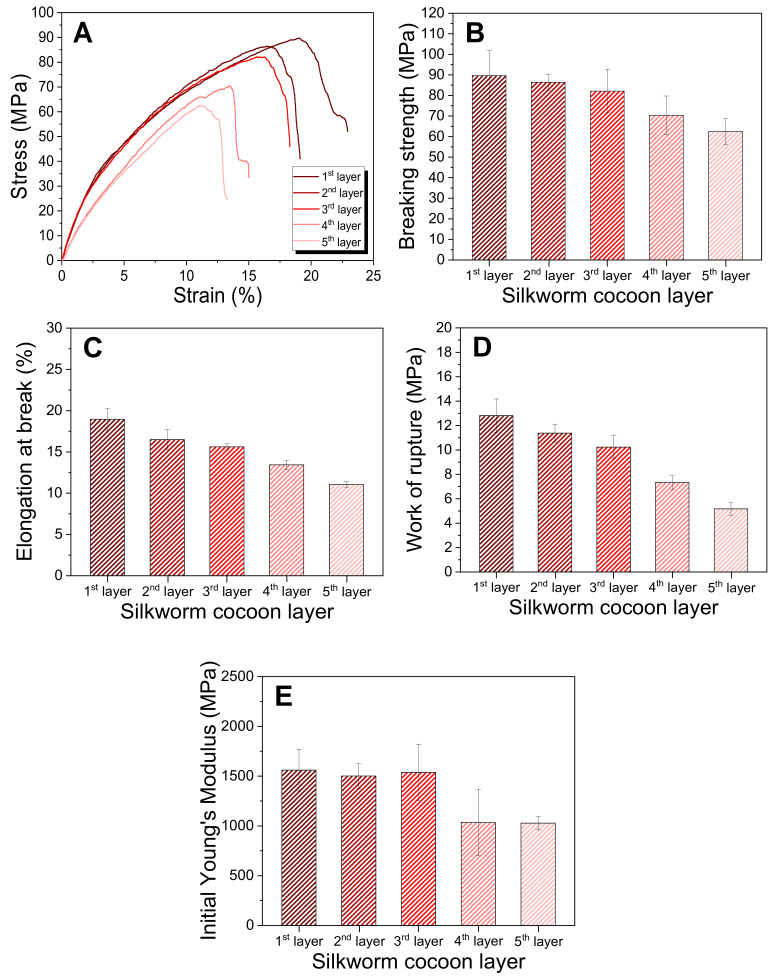
(**A**) Representative stress–strain curve, (**B**) breaking strength, (**C**) elongation at break, (**D**) work of rupture, and (**E**) initial Young’s modulus of the nonwoven silk fabrics prepared with fibers from different silk cocoon layers (*n* = 5).

Figure 7 shows the mechanical test result for the nonwoven silk fabric prepared using fibers from different cocoon layers. As the number of layers increased, the tensile strength, elongation at break, and work of rupture of the nonwoven silk fabric decreased. Notably, the mechanical properties of the nonwoven silk fabric deteriorate with the increasing content of the highly crystalline component (i.e., silk fibroin). This can be ascribed to the binding effect of sericin, which overwhelmed the effect of the more crystalline silk fibroin; that is, the nonwoven silk fabric is fabricated by the binding ability of sericin [29,30,31,32,33,34,35]. As a result, the mechanical properties of the nonwoven silk fabric are improved by increasing amount of sericin [32,34,35]. Hence, this suggests that the binding effect of sericin overwhelms the effect of fibroin on enhancing the mechanical properties of the nonwoven silk fabric.

### 2.4. Cell Viability of the Nonwoven Silk Fabric

**Figure 8:**
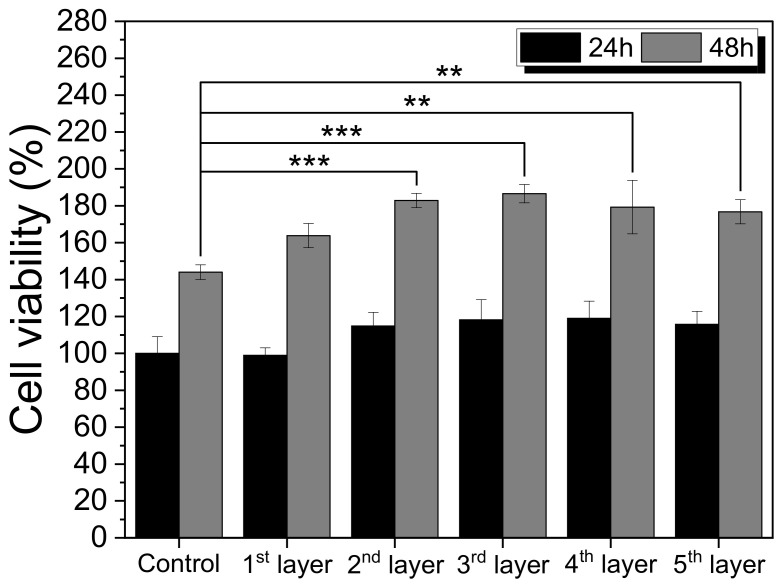
Cell viability of the nonwoven silk fabrics prepared with different silk cocoon layers (** *p* < 0.01, *** *p* < 0.001).

Cell viability is an important material property for biomedical and cosmetic applications [14,15,16,34,60]. Figure 8 shows the cell viability of the nonwoven silk fabric prepared with fibers from different cocoon layers. With an incubation time of 24 h, no significant difference is observed between the control and nonwoven silk fabrics. With an incubation time of 48 h, the nonwoven silk fabric prepared with fibers from the 1st layer is similar to that of the control, whereas those from the 2nd and 3rd layers (*** *p* < 0.001) and 4th and 5th layers (** *p* < 0.01) have significant differences with the control. Meanwhile, no significant difference was observed among the use of the five layers.

The cell image of the nonwoven silk fabrics (Table 4) confirms the Cell Counting Kit (CCK) test results in Figure 8. As the incubation period is increased to 48 h, more viable cells are observed. Regardless of the cocoon layer used, all nonwoven silk fabrics have comparable number of live cells to that of the control, indicating the cytocompatibility of the nonwoven silk fabrics with fibers from different cocoon layers. Considering that the nonwoven silk fabric is composed of natural silk fibers, their good cell viability is consistent with that of silk, as reported in previous studies [11,12,34,35,60].

## 3. Materials and Methods

### 3.1. Materials

*Bombyx mori* Baekokjam silkworm cocoons were provided by Gyeongsangbuk-do Silkworm & Insect Management Center (Sangju, Republic of Korea). To minimize the variation of weight and fiber amount of silkworm cocoon, we selected 0.7~0.8 g silkworm cocoons and used them for the preparation of silk web and nonwoven silk fabric.

### 3.2. Preparation of the Silk Web and Nonwoven Silk Fabric

**Figure 9:**
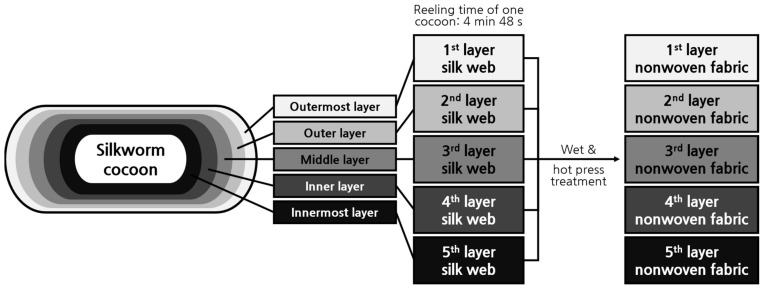
Schematic for the manufacturing of five different types of silk web and nonwoven silk fabric using silk fibers from different silkworm cocoon layers.

Five different types of silk web and nonwoven silk fabric were prepared with silk fibers from five different layers of silkworm cocoons (Figure 9). We divided the layers evenly based on the length of the silk fiber in the silkworm cocoon; that is, the reeling speed was controlled to a constant value of 66.5 cm/s for all samples. The average time required to reel silk fibers in the silkworm cocoons was 24 min. Therefore, the reeling time for each layer in the cocoon was fixed to 4.8 min. The five layers are referred to as the 1st layer (outermost layer), 2nd layer (outer layer), 3rd layer (middle layer), 4th layer (inner layer), and 5th layer (innermost layer).

**Figure 10:**
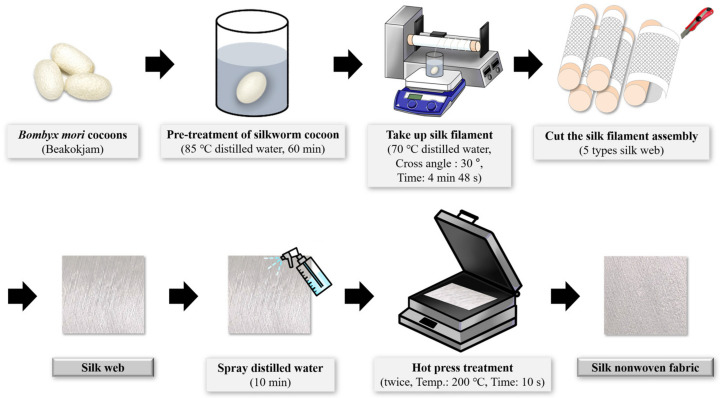
Schematic of the fabrication process of the silk web and nonwoven silk fabric.

The fabrication process of the silk web and nonwoven silk fabric, as reported in previous studies [29,30,31,33], is presented in Figure 10. First, as a pretreatment, the Baekokjam silkworm cocoons were immersed in a bath of distilled water at 85 °C for 60 min for the swelling of silk sericin. The silkworm cocoons were then moved to a reeling bath at 70 °C. The silk web was produced by reeling silk filaments into the winder of the Silk Web Manufacturing Machine (SNWFM-1, Donga Machinery, Namyangju, Republic of Korea). The reeling and transverse speeds were fixed to 66.5 and 19 cm/s, respectively, to create a cross angle of 30° between the silk filaments [29]. After winding 50 silkworm cocoons for each layer, the silk webs were cut and separated from the bobbin of the winder.

To produce nonwoven silk fabric, the silk web was moisturized by spraying distilled water with a sprayer for 10 min and then pressed twice using a hot presser (HK 2008-1-5, Hankuk Industry Co., Gwangju, Republic of Korea) at 200 °C for 10 s. To prevent the silk web from adhering to the hot press plates, polyester nonwoven fabrics were placed above and below the silk web [29,30,31,32,33,34,35]. After the hot press method, nonwoven silk fabrics were obtained by removing the polyester nonwoven fabrics.

### 3.3. Measurement and Characterization

The morphologies of the silk web and nonwoven silk fabric were examined by field-emission scanning electron microscopy (FE-SEM, S-4800, Hitachi, Tokyo, Japan) with secondary electron detector. The samples were coated with Pt–Pd before the observation, and the acceleration voltage was 5.0 kV [31,61]. The means and standard deviations of the diameters of the silk filaments in the web were obtained by measuring 50 silk filaments from the SEM image analysis program (DIMIS-PRO 2.0, Siwon Optical Technology, Anyang, Republic of Korea).

The thicknesses of the silk web and nonwoven silk fabric were measured using a thickness gauge (MDC-25PXT, Mitutoyo, Kawasaki, Japan) at 21 different locations. The means and standard deviations of the thicknesses were then calculated based on the measurements.

The amino acid composition of the nonwoven silk fabric was analyzed using an amino acid autoanalyzer (L-8900, Hitachi, Tokyo, Japan). Each sample was hydrolyzed with 6 N HCl and passed through an ion-exchange column to separate the amino acids. When the amino acids separated from the column reacted with ninhydrin at a high temperature (135 °C) to form a chromogenic compound, the absorbance of the compounds was measured at two wavelengths (570 and 440 nm) [12,62].

The color of the silk web and nonwoven silk fabric was examined by CIE 1931 color space. CIE tristimulus (XYZ) values were based on the CIE standard illuminant D65 with the specular-component-excluded mode of the colorimeter (Konica Minolta, CR-300 Chroma meter, Osaka, Japan). The yellowness index was calculated using Equation (1) [63].

(1) $$\mathrm{Yellowness}\;\mathrm{index}\;\left(\%\right)=\frac{\left(1.28X-1.06Z\right)}{Y}\times 100$$

The molecular conformation of the silk web and nonwoven silk fabric was examined by FTIR (Nicolet 380, Thermo Fisher Scientific, Waltham, MA, USA) through the ATR method (Smart iTR ZnSe). The scan range, scan number, and resolution were 4000–650 cm^−1^, 32, and 8 cm^−1^, respectively [30,33]. The proportions of the β-sheet and random coil conformation were determined by deconvoluting the amide I band (1600–1700 cm^−1^) using the Fourier self-deconvolution fitting method in the Origin 8.0 software to examine the differences in the silk web and nonwoven silk fabric prepared with fibers from different silk cocoon layers [35,64,65].

The crystalline structure of the silk web and nonwoven silk fabric was determined using a wide-angle X-ray scattering system (D8 Discover, Bruker, Karlsruhe, Germany) using Cu Kα radiation. The irradiation conditions were 50 kV and 1000 μA, and the measurement time was 600 s.

To determine the moisture regain of the silk web, the samples were kept under standard conditions (20 °C and 65% relative humidity) for 24 h. The moisture regain of the silk web was calculated using Equation (2). The dry weight of the silk samples was determined with a moisture-balance instrument (XM60, Precisa Gravimetrics, Dietikon, Switzerland) [44].

(2) $$\mathrm{Moisture}\;\mathrm{regain}\;\left(\%\right)=\frac{\mathrm{Initial}\;\mathrm{weight}-\mathrm{Dry}\;\mathrm{weight}}{\mathrm{Dry}\;\mathrm{weight}}\times 100$$

The water contact angle was measured by the sessile drop procedure using a contact angle meter (Dino-Lite, AM703MZT, Seoul, Republic of Korea). The samples were cut into 2 × 2 cm^2^ pieces, and 10 μL of distilled water was placed on each sample at room temperature. Images were obtained at 30 s after the liquid was dropped, and the contact angle was calculated from the images.

DSC analysis was performed using a thermal analysis instrument Q10 (DS25, TA Instrument, New Castle, DE, USA) in the range of 60–270 °C at a scanning rate of 10 °C/min. The analysis was carried out under 50 mL/min nitrogen gas [59].

The mechanical properties of the nonwoven silk fabrics were evaluated using a universal testing machine (OTT-003, Oriental TM, Ansan, Republic of Korea) with a load of 200 kgf and extension rate of 10 mm/min. The gauge length was 30 mm. The samples were cut into 50 × 10 mm^2^ pieces and preconditioned at 20 °C and 65% RH. Seven samples were tested for each condition, and the average and standard deviation of the measurement results were calculated from five results after the maximum and minimum values were removed.

L929 cells were grown in a RPMI1640 medium (Gibco, Billings, MT, USA) supplemented with 10% (*v*/*v*) fetal bovine serum and 1% (*v*/*v*) antibiotic–antimytotic solution. The L929 cells were incubated at 37 °C in a humidified 5% CO_2_ atmosphere. When 80% confluence was observed, the subculture was performed twice per week.

In vitro cytotoxicity test was performed by employing an extract method in accordance with ISO 10993-5. Before extraction, each sample was sterilized with ethylene oxide gas. The extraction was performed by immersing the samples (6 × 3 cm^2^) in 6 mL RPMI1640 culture medium with gentle shaking at 37 °C for 24 h. The ratio of the sample surface area to the extraction vehicle volume was 3 cm^2^/mL. The cytotoxicity of the samples for the L929 cells was determined by applying the CCK-8 (Cell Counting Kit 8, Dojindo, Japan) assay in vitro. The L929 cells were seeded into 96-well plates at 1 × 10⁴ cells/well and incubated at 37 °C for 24 h in a 5% CO_2_ atmosphere. The culture medium was then replaced with 100 µL/well sample extracts. After 24 and 48 h of incubation, the extracts were discarded for the CCK assay, and 100 µL 10% (*v*/*v*) CCK-8 solution was added to each well. After incubation for 1 h, the absorbance was measured at 450 nm. Subsequently, cell viability of sericin was calculated using Equation (3) [66].

(3) $$\mathrm{Cell}\;\mathrm{viability}\;\left(\%\right)=\frac{{\mathrm{OD}}_{\exp}-{\mathrm{OD}}_{\mathrm{blank}}}{{\mathrm{OD}}_{\mathrm{control}}-{\mathrm{OD}}_{\mathrm{blank}}}\times 100$$

Cytotoxicity was evaluated by performing fluorescence staining using a live/dead viability/cytotoxicity kit (L3224, Invitrogen, Waltham, MA, USA), as per the manufacturer’s protocol. The L929 cells were seeded into a 24-well plate at 3 × 10⁴ cells/well and incubated for 24 h under a 5% CO_2_ atmosphere at 37 °C. Subsequently, the culture media were replaced by 300 µL/well sample extracts. After 24 and 48 h of incubation, the extracts were discarded, and 300 µL staining solution was added to each well. After incubation for 45 min, the staining solutions were removed, and the cells were observed using a fluorescence-inverted microscope (IX83, Olympus, Tokyo, Japan).

## 4. Conclusions

In this study, silk fibers from five different layers of silkworm cocoon were reeled to fabricate five silk webs and five nonwoven silk fabrics. Subsequently, their structural characteristics and properties were examined. The diameter of the silk fiber and thickness of the nonwoven silk fabric decreased using fibers from the outermost to the innermost layer. The hydrophilicity and crystallinity of the silk web and nonwoven silk fabric increased using fibers from the outermost to the innermost layer. The nonwoven silk fabric with fibers from the outer layers exhibited better mechanical properties than that with the fibers from the inner layers.

Overall, the key finding of this study is the diverse structure and properties of silk webs and nonwoven silk fabrics obtained using silk fibers from different layers of silkworm cocoon. Considering their different performances for cosmetic and biomedical applications, the findings in this work can be effectively utilized to increase the applicability of silk webs and nonwoven fabrics in bio-related industries.

## Figures and Tables

**Table 1:** FE-SEM images of the silk web and nonwoven silk fabric prepared with fibers from different cocoon layers. The magnification bars represent 100 μm.

	1st Layer	2nd Layer	3rd Layer	4th Layer	5th Layer
Silk web	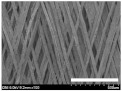	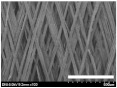	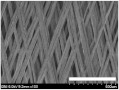	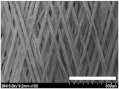	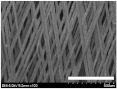
Nonwoven fabric	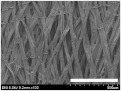	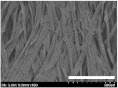	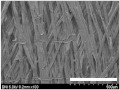	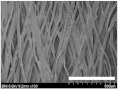	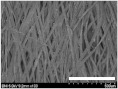

**Table 2:** Amino acid composition of the nonwoven silk fabrics prepared with fibers from different silk cocoon layers.

Nonwoven Fabric	1st Layer	2nd Layer	3rd Layer	4th Layer	5th Layer
Amino Acid (mol %)					
Aspartic acid	5.55	4.46	3.96	3.69	3.55
Threonine	3.05	2.49	2.23	2.09	1.95
Serine	14.19	12.63	12.05	11.74	11.36
Glutamic acid	2.11	1.81	1.70	1.61	1.66
Glycine	35.92	38.37	39.82	40.00	40.30
Alanine	23.78	25.96	26.54	27.71	27.83
Cysteine	0.57	0.52	0.38	0.32	0.49
Valine	2.62	2.52	2.48	2.41	2.38
Methionine	0.16	0.14	0.13	0.12	0.12
Isoleucine	0.70	0.67	0.65	0.62	0.63
Leucine	0.70	0.62	0.59	0.55	0.56
Tyrosine	4.36	4.50	4.53	4.54	4.58
Phenylalanine	0.78	0.77	0.77	0.74	0.75
Lysine	1.23	0.94	0.81	0.74	0.75
Histidine	0.58	0.46	0.40	0.38	0.38
Arginine	1.53	1.29	1.19	1.08	1.02
Proline	2.17	1.85	1.77	1.66	1.69
Total	100.00	100.00	100.00	100.00	100.00

**Table 3:** XRD patterns of the fibers of the silk web and nonwoven silk fabric prepared with fibers from different silk cocoon layers.

	1st Layer	2nd Layer	3rd Layer	4th Layer	5th Layer
Silk web	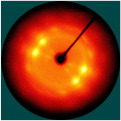	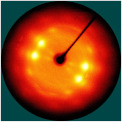	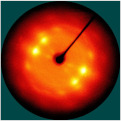	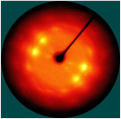	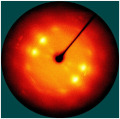
Nonwoven silk fabric	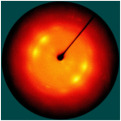	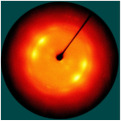	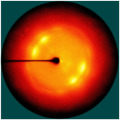	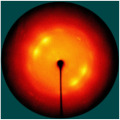	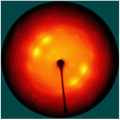

**Table 4:** Fluorescence images of the cell viability assay of the nonwoven silk fabrics prepared using fibers from different silk cocoon layers. The white bars represent 200 μm.

	Control	1st Layer	2nd Layer	3rd Layer	4th Layer	5th Layer
24 h	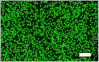	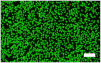	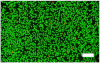	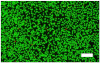	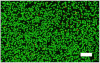	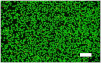
48 h	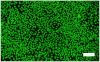	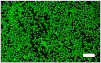	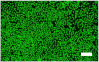	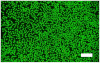	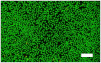	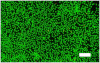

## Data Availability

The data presented in this study are available on request from the corresponding author.
